# Cognitive load theory in workplace-based learning from the viewpoint of nursing students: application of a path analysis

**DOI:** 10.1186/s12909-024-05664-z

**Published:** 2024-06-18

**Authors:** Shakiba Sadat Tabatabaee, Sara Jambarsang, Fatemeh Keshmiri

**Affiliations:** 1 Kish International Campus, University of Tehran, Tehran, Iran; 2 Center for Healthcare Data Modeling, Department of Biostatistics and Epidemiology, School of Public Health, Shahid Sadoughi University of Medical Sciences, Yazd, Iran; 3 Department of Medical Education, Education Development Center, Shahid Sadoughi University of Medical Sciences, Yazd, Iran

**Keywords:** Cognitive load, Memory, Learning, Decision-making, Extraneous cognitive load, Workplace- based learning, Clinical education, Nursing, Intrinsic cognitive load

## Abstract

**Purpose:**

The present study aimed to test the relationship between the components of the Cognitive Load Theory (CLT) including memory, intrinsic and extraneous cognitive load in workplace-based learning in a clinical setting, and decision-making skills of nursing students.

**Methods:**

This study was conducted at Shahid Sadoughi University of Medical Sciences in 2021–2023. The participants were 151 nursing students who studied their apprenticeship courses in the teaching hospitals. The three basic components of the cognitive load model, including working memory, cognitive load, and decision-making as the outcome of learning, were investigated in this study. Wechsler’s computerized working memory test was used to evaluate working memory. Cognitive Load Inventory for Handoffs including nine questions in three categories of intrinsic cognitive load, extraneous cognitive load, and germane cognitive load was used. The clinical decision-making skills of the participants were evaluated using a 24-question inventory by Lowry et al. based on a 5-point scale. The path analysis of AMOS 22 software was used to examine the relationships between components and test the model.

**Findings:**

In this study, the goodness of fit of the model based on the cognitive load theory was reported (GIF = 0.99, CFI = 0.99, RMSEA = 0.03). The results of regression analysis showed that the scores of decision-making skills in nursing students were significantly related to extraneous cognitive load scores (*p*-value = 0.0001). Intrinsic cognitive load was significantly different from the point of view of nursing students in different academic years (*p* = 0.0001).

**Conclusion:**

The present results showed that the CLT in workplace-based learning has a goodness of fit with the components of memory, intrinsic cognitive load, extraneous cognitive load, and clinical decision-making skill as the key learning outcomes in nursing education. The results showed that the relationship between nursing students’ decision-making skills and extraneous cognitive load is stronger than its relationship with intrinsic cognitive load and memory Workplace-based learning programs in nursing that aim to improve students’ decision-making skills are suggested to manage extraneous cognitive load by incorporating cognitive load principles into the instructional design of clinical education.

## Introduction

Cognitive load was introduced as a key theory in medical education [1] This theory guides the components of human cognitive architecture concerning learning and education to create a correct understanding of the characteristics and conditions of education and learning [2].

### Cognitive load theory (CLT)

The CLT was first proposed in the 1980s by John Sweller [3]. This theory explains learning according to three important aspects including types of memory (working and long-term memory), learning process, and forms of cognitive load that affect learning [4].

#### Memory

The cognitive architecture assumed by CLT includes long-term memory (LTM) and working memory (WM). The key subsystem of memory in the CLT is working memory [5].

#### Cognitive load

Cognitive load is defined as the load that a specific task imposes on the learner’s cognitive system [6]. In the CLT, three types of cognitive load are proposed, including intrinsic cognitive load (ICL), extraneous cognitive load (ECL), and germane cognitive load (GCL) [7, 8]. ICL is related to the complexity of educational materials rather than their quantity [9]. ICL depends on several factors, including the individual’s skill, the number of information elements, and the degree of interaction of different elements of the tasks. ECL caused by the training format includes training strategies, training design, and teaching-learning methods [4, 10, 11]. GCL refers to the load imposed by the mental processes necessary for learning (such as the formation of schemata) [11]. Germane load means trying to build and modify learning schemata, which is mainly under the control of job components such as motivation, effort, and the learner’s metacognitive skills [7]. Also, the level of learner’s proficiency can moderate the ICL arising from the interaction of elements. This means that the availability and automaticity of the learner’s schemata can moderate intrinsic load [11].

#### Learning process

Education in medical science systems is a complex and multidimensional process that is affected by many factors [12]. In the process of clinical education, students need to learn several professional tasks and activities and apply them in the provision of health care services by simultaneously integrating a set of knowledge, skills, and behaviors [11]. These characteristics of clinical education can impose a high cognitive load on students and harm their effective learning [11, 13].

### CLT in health professions education

The CLT has emerged as one of the foremost models in educational psychology considered in different fields such as health professions education. The goal of CLT has been to improve learning at the individual student level in different environments including the classroom, and complex professional learning environments [14]. Sweller and colleagues showed there have been main developments in CLT and instructional design over the last 20 years. The ‘cognitive theory of multimedia learning’ focusing on the design of multimedia educational materials and the ‘four-component instructional design (4 C/ID)’ focusing on the design of whole-task courses and curricula have been built based on the CLT [15]. In addition, the CLT provides principles that are recommended to apply to the design of instructional messages and instructional units, such as lessons, written materials consisting of text and pictures, and educational multimedia (instructional animations, videos, simulations, games) [15].

The theoretical scope of the cognitive load has been expanded by including the physical environment as a key factor affecting cognitive load. Physical environments that evoke stress, emotions, and/or uncertainty raise new questions about how to deal with cognitive load. The questions require examining the human cognitive architecture of educational design in environments that are accompanied by uncertainty and stress [15]. Likewise, Paas et al. (2020) introduced variables affecting cognitive load and introduced factors including instructional design and learning environment as an effective factor that affects students’ learning process. They stated that the learning environment can affect cognitive load and suggested a way of managing it [5].

Advances in CLT have set the trends for future developments in different learning environments such as workplace-based learning, simulation, and games [15]. Most studies used the CLT principles in instructional design in simulation, virtual reality, and game settings in nursing education [1, 16–18]. Yiin et al., (2023) indicated the multi-media interactive learning materials and an active learning mechanism reduced nursing students’ intrinsic and extrinsic cognitive load and encouraged the students to learn [19]. Takhdat et al., (2024) showed that mindfulness meditation practice optimizes cognitive load, and decreases the anxiety of nursing students in a simulation setting [20].

Clinical education in the workplace is defined as a main educational setting where students improve their competencies and prepare for their future careers. Sewell and colleagues (2019) in a BEME guide (Best Evidence in Medical Education guide) discussed cognitive load in workplace-based learning in the real environment [21]. The workplace-based learning in clinical education imposes high levels of cognitive load that negatively impact on learning of learners and their performances. Sewell et al. indicated the factors of, complex tasks, settings, and novice learners mostly predispose the students to high levels of cognitive load. They stated aspects of workplace environments contribute to extraneous load, and adversely impact capacity for engaging in tasks that enhance germane load and learning [15]. Further studies are recommended to understand the manner and the extent of the impact of cognitive load on different learning outcomes in various learning environments in systems of health professions education [1, 16, 17].

The present study aimed to test the relationship between the components of the Cognitive Load Theory (CLT) including memory, intrinsic and extraneous cognitive load in workplace-based learning, and decision-making skills of nursing students in clinical settings.

## Materials and methods

This cross-sectional study was conducted in 2021–2023 at Shahid Sadoughi University of Medical Sciences, Yazd, Iran. In the present study, the path analysis was used to predict a defined theoretical model that posits hypothesized linear relations among variables and decreases to the solution of one or more multiple regression analyses.

### Setting

The present university has conducted a four-year nursing degree curriculum. The students have participated in workplace-based learning in the clinical setting from the second semester. They contributed to care processes as team members from the third semester of the academic course. In the present nursing curriculum, there is no reasoning and decision-making training course. The decision-making skills have been learned by the students in the process of workplace-based learning in the clinical environment. The stages of experiential learning, including observation, practice and repetition, feedback, and self-reflection, have been implemented in the nursing clinical education program. In clinical education courses, the students have used study guides nursing flowcharts, and clinical guidelines.

### Participants

Undergraduate nursing students of the faculties affiliated with Shahid Sadoughi University of Medical Sciences participated in this study. The inclusion criteria were nursing students who had completed at least six months of apprenticeship courses in their field in the hospital. Students with working experience as health technicians (*Behvarz*) were excluded from the study. This exclusion criterion aims to control for potential confounding variables that could influence the study’s outcomes, such as previous professional experience impacting cognitive load assessments and decision-making skills [22, 23].

The rule of thumb is to have at least 10–15 observations per parameter (i.e., 10–15 cases for each independent variable and the dependent variable) to have reliable estimates of the model parameters [24]. Thus, a total of 151 eligible students were randomly selected in this study.

### Data collection

To conduct the examination, the researcher explained the objectives of the research, the instruments of data collection, the duration of the examinations, and the confidentiality of data. The participants were asked to perform the Wechsler computerized working memory test and fill the Questionnaires of Cognitive Load Inventory for Handoffs and Clinical Decision-making in a calm environment and away from disturbing side factors. The informed consent form was completed by the students.

### Study tools

Working memory measurement tool: Wechsler’s computerized working memory test was used to evaluate working (active) memory [25, 26]. In this test, two sections of forward and backward recall of digits are used to measure the memory span. The total working memory score is obtained from the sum of the scores of the two parts of forward and backward recall with a maximum score is 28. For the correct evaluation of the subject, the soft table is used for the desired ages. In this software, the score of memory span (auditory and visual) is also provided. This score represents the number of items memorized by the examinee.

The cognitive Load Inventory for Handoffs (CLIH) was compiled by Yang et al., (2016) [27] to assess the cognitive load of students in their clinical education. The questionnaire includes 9 questions in three domains of ICL, ECL, and GCL which is based on a 10-point Likert. The validity of the tool was confirmed in the present study. The qualitative content validity of the Persian version of the questionnaire was confirmed from the viewpoints of 15 experts. To determine content validity quantitatively, two indices “Content Validity Ratio (CVR)” and “Content Validity Index (CVI)” were used. The findings of the quantitative content validity assessment indicated that the CVR for all items was higher than the minimum acceptable value (= 0.49), and the CVI values of all items were above 0.79. According to the indices, all items were kept in the questionnaire. S-CVI/Ave was 0.94, which was desirable. The internal consistency of the tool was reported as Cronbach’s alpha coefficient = 0.86.

Clinical decision-making as a learning outcome of nursing students in clinical education was evaluated using the 24-item questionnaire designed by Lauri et al. (2001) which is based on a 5-point scale [12]. The reliability and validity were confirmed in the Karimi et al. study (2013) (Cronbach’s alpha coefficient of intrinsic consistency = 0.8) [28].

### Data analysis

Demographic information of the participants (including gender, age, level of education, and the last externship/internship period of the students) was collected. Descriptive statistics (including frequency percentage, mean, and standard deviation) and analytical statistics (ANOVA) were used to investigate the variables. SPSS statistical software (Ver. 24) was used for data analysis.

This study employed path analysis as the primary statistical analysis method due to its ability to examine the relationships between multiple variables, including the direct and indirect effects of predictor variables on the outcome variable. Specifically, path analysis was used to investigate the relationships between memory, internal and external cognitive load, and decision-making skill, as well as the indirect effects of these variables on learning outcomes. Moreover, path analysis is suited for examining the relationships among the variables in this study due to the capability of path analysis to handle complex models and multiple relationships simultaneously. The use of path analysis was further justified by the need to examine the causal relationships between variables, as well as to account for measurement error and unexplained variance in the data. Path analysis allows for the estimation of standardized regression coefficients, which can be used to interpret the magnitude and direction of the relationships between variables.

In terms of model evaluation, this study employed several indices to assess the goodness-of-fit of the proposed model. The goodness-of-fit index (GFI) was also used to evaluate the model’s fit relative to a baseline model, with a value of 0.95 or higher indicating a good fit [29]. In addition, acceptable levels of indices of the path analysis include Adjusted Goodness-of-Fit Index (AGFI) > 0.8, Tucker-Lewis Index (TLI) > 0.9, the Incremental Fit Index (IFI) > 0.8. Regarding the Comparative Fit Index (CFI) with a value of greater than 0.90 is very good fit, 0.80 to 0.89 is adequate but marginal fit, 0.60 to 0.79 is poor fit, a and lower than 0.60 very poor fit. Finally, the root mean square error of approximation (RMSEA) was used to evaluate the model’s fit to the data, with a value of 0.05 or less indicating a good fit [30]. These results indicate that the proposed model provided an adequate representation of the relationships among the variables studied. In the present study, AMOS 22 software was used to assess the fitness of this model.

## Findings

In total, 151 nursing students participated in this study, 77 of them (51%) were women and 74 (49%) were men. The mean age of the participants was 21.97 ± 2.20. The demographic information of the participants is shown in Table 1.

**Table 1:** Demographic information of the participants

		Number	Percent
Gender	Female	77	51
	Male	74	49
**Variable classification**			
Academic year	2	62	41.05
	3	32	21.19
	4	57	37.74
GPA*	A (18–20)	21	13.9
	B (16–18)	84	55.6
	C (14–16)	44	29.1
	D (< 14)	2	1.3

*Grade Point Average (GPA)

The mean score of decision-making of nursing students was 78.37 ± 11.30 and the mean score of cognitive load perceived by students in the workplace-based learning process of clinical setting was 45.26 ± 8.84. Table 2 shows the mean score of the students in the studied variables.

**Table 2:** The mean score of the students in the studied variable

	Minimum	Maximum	Mean	Std. Deviation
Decision- making skill	27.00	121.00	78.37	11.30
ICL*	4.00	37.00	19.85	6.52
ECL**	6.00	26.00	16.07	3.89
Total scores of cognitive load	24.00	118.00	45.94	11.32

* ICL: Intrinsic cognitive load

** ECL: Extraneous cognitive load

The results of regression analysis showed that the students’ scores of nursing students in decision-making skills were significantly related to the ECL scores (*P* = 0.0001). By increasing one ECL score, the score of students’ clinical decision-making skill increased by 1.2.

**Fig. 1:**
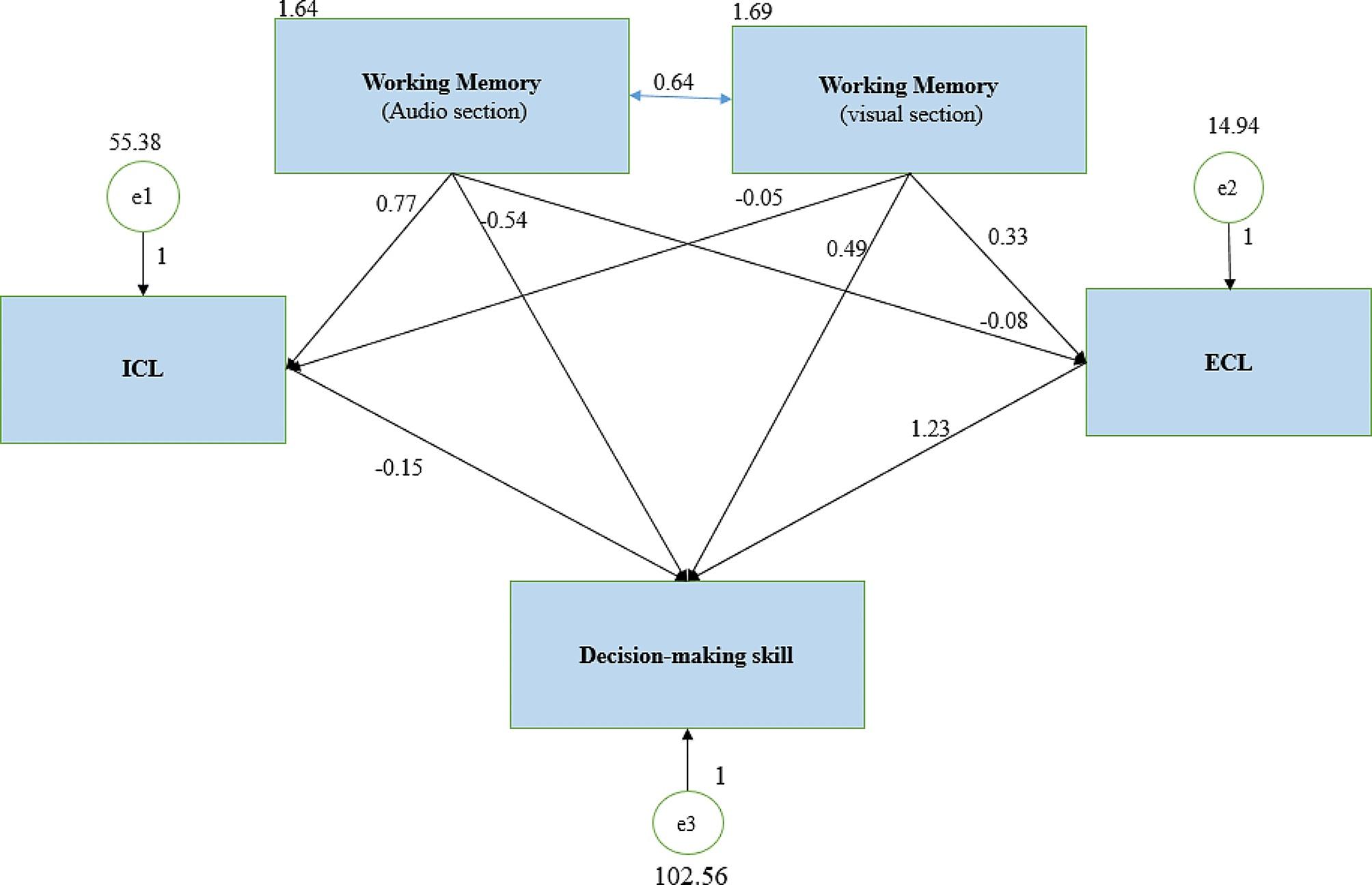
Path analysis model: standardized coefficient estimates ICL: Intrinsic cognitive load, ECL: Extraneous cognitive load

The mean scores of ICL and ECL of the students according to their academic year are reported in Table 3. ANOVA showed that ICL was significantly different from the point of view of nursing students in different academic years (*P* = 0.0001). The results of the Bonferroni test showed that ICL in novice (second-year) students was significantly lower than in third-year (*P* = 0.0001) and fourth-year students (*P* = 0.004). Figure 1 illustrate the path analysis model of CLT in the workplace-based leaning. Table 4 show a report of indices of goodness-of-fit in the model.

**Table 3:** The ICL and ECL scores of the students in different academic years

		Mean	Std. Deviation	95% Confidence Interval for Mean		Minimum	Maximum
				Lower Bound	Upper Bound		
ICL	2nd year	17.14	5.52	15.74	18.54	4.00	30.00
	3rd year	23.46	7.03	20.93	26.00	10.00	37.00
	4rd year	20.77	6.07	19.15	22.38	6.00	37.00
	Total	19.85	6.52	18.80	20.90	4.00	37.00
ECL	2nd year	16.53	3.47	15.64	17.41	9.00	26.00
	3rd year	16.37	4.48	14.75	17.99	7.00	26.00
	4rd year	15.40	3.95	14.35	16.45	6.00	26.00
	Total	16.07	3.89	15.44	16.69	6.00	26.00

**Table 4:** Summary of model fit statistics

	CFI	GFI	TLI	RMSEA	AGFI	IFI
Model 1	0.997	0.997	0.973	0.030	0.955	0.998

CFI = Comparative Fit Index, GFI = Goodness of fit index, TLI = Tucker-Lewis Index, RMSEA = Root Mean Square Error of Approximation, AGFI = Adjusted Goodness-of-Fit Index, IFI = the incremental fit index

## Discussion

The current study reported a statistically significant fit for the proposed path analysis model indicating a good fit in the data collected from the nursing students in the workplace-based learning at clinical setting.

The development of clinical decision-making skills is a main competency of nursing students in clinical education courses. Learning the decision-making skill is considered a complex and multi-dimensional process that is influenced by various factors for instance personal features, task experience, and situational awareness ability [22]. Moreover, educational factors such as instructional design, learning environments, and teaching methods direct the cognitive load and learning process of students [12, 31]. The present results showed that nursing students’ decision-making skills have a significant positive relationship the capacity of the working memory of learners and ECL in workplace-based learning environments. In line with our results, the findings of studies confirmed management of ECL that depended on the characteristics of the instructional material, the instructional design, and the prior knowledge of learners in the process of clinical education have a positive relationship with learning [5, 21, 23]. The effect of cognitive load as a mediating relationship on clinical reasoning as the key outcome of learning was shown in the Jung et al. study (2022) [32]. In a review, Josephsen et al. (2015) showed that there is a positive relationship between the cognitive architecture of learners and educational design in nursing. Their results indicated that learners must be aware of cognitive architecture and educational processes in nursing to manage cognitive load and effective learning [16].

The present results showed that the decision-making scores of the nursing students had a significant positive relationship with ECL in workplace-bead learning. The students have experienced the experiential learning process in clinical nursing education. They learned through observing, exercising, receiving feedback, and reflecting in action and on action at the workplace-based learning in the clinical setting. In addition, nursing students used supportive resources such as a nursing flowchart, a study guide, and structured constructive feedback in clinical education. The use of CLT principles in the instructional design of workplace-based learning of nursing clinical education effects on the ECL. Many learning tasks, especially complex clinical activities, require memorizing and applying a large amount of information [11]. According to the CLT, the educational environment provides a trigger to use the information stored in LTM to determine the appropriate action in the environment according to the environmental-and-organizing linking principle. Moreover, specialized performance is developed through the creation of a large number of more complex schemata by combining elements consisting of lower-level schemata with higher-level schemata [5]. The schemata facilitate the decision-making process. The significant relationship between the ECL and learning has also been confirmed in the study of Sawicka et al. (2008) [9]. The application of strategies for ECL management is recommended by Sawicka et al. The tailored strategies with the workplace-based education were conducted in the clinical setting. These strategies include presenting educational materials from simple to complex and presenting familiar examples in the experiential learning process in clinical setting. The students were experienced the nursing care plan form simple cases to complex cases. The supplementary questions and diverse assignments were conducted in the clinical education by students. They experienced self-explanatory and supporting information in the feedback and reflection process. The use of the strategies in the clinical education of the nursing students in workplace-based learning may effect on our findings. Similarly, Skulmowski et al., (2022) acknowledged the use of aspects of constructive alignment, a strategy to balance the cognitive load and an approach of fostering deep forms of learning improved the learning outcomes [33].

In the CLT, the features of working memory including its capacity and time limitations were introduced as a key component that plays an important role in learning. This issue is emphasized in cognitive models [5, 34]. The present results showed that the relationship between memory and ICL is stronger than ECL. Kilic et al. (2010) model indicated that working memory plays an effective role in providing information necessary for complex cognitive activities such as learning and clinical reasoning [34]. So, if the learning material is too difficult, the ICL imposed on learners may exceed their working memory capacity and hinder learning [9]. In line with our results, the relationship of working memory with ICL was stranger than ECL. Sawicka (2008) stated that the insufficiency of working memory resources to expand schemata hinders learning [9].

The present results showed that the fit of the model was favorable by considering working memory scores, cognitive load, and learning, but no significant relationship was observed between working memory and decision-making scores. Also, no significant relationship between ICL and learning was observed in the present study. In line with our results, Szulewski et al., (2021) presented a new model for medical education systems based on CLT. They stated the relationship between the working memory of healthcare workers cannot be discussed directly in the model. They expressed this as a limitation of their model and acknowledged that the capacity of working memory in complex medical education systems is affected by stress, emotions, and uncertainties, which can affect the performance of healthcare workers [14]. Although the significant relationship between the components was not approved in the present study, the good fit of the proposed path analysis model, indicated that these components interact with each other and require consideration as a coherent structure in instructional design of the workplace-based learning by planners.

Emotions, stress, and uncertainty are integrated with the learning process and environment in the educational systems of health professions. The educational systems of health professions integrate emotions, stress, and uncertainty into the learning process and environment. According to Sweller, emotions that are considered undesirable for learning result in extraneous load that can be reduced by preventing them. If emotion, stress, and uncertainty are seen as an integral element of the task that learners require to learn, they contribute to intrinsic cognitive load and must be dealt with in another way. Therefore, it is necessary to consider multi-faceted planning by using different components and systematically examining different aspects of cognitive load before formulating educational designs for workplace-based learning in the clinical setting [5].

Garvey et al. study (2017) introduced a model in which, in addition to the cognitive load components, the individual maturity component based on the years of education was also included in the model [35]. In the present study, individual maturity was considered in different academic years. The present results showed that there is a significant relationship between the learning maturity of individuals and ICL components. ICL is related to the complexity caused by training and depends on factors such as the individual’s skill, the number of information elements, and the degree of interaction of elements in the learning process. Our findings indicated the ICL of the second-year students was significantly lower compared to the third-year and the fourth-year. The results can be due to less work experience in the hospital, the smaller amount of material learned, and dealing with the limited clinical complexities of the students in the second year. Sewell’s results confirmed a negative relationship between GCL and ICL with the level of experience and performance of students [21]. These results were also aligned with the present results. Our results are in contrast to Schlairet’s findings (2015) which indicated that a negative relationship between the performance of novice nursing students and cognitive load was observed, although this relationship was not significant [36]. The difference in the level of students and the difference in the measured learning outcome (decision-making skills versus performance) and considering the cognitive load score without separating ICL and ECL can affect the results.

The results showed that the current model does not have a good fit considering the GCL. The current limitation can be due to measuring the GCL using only one question in CLIH [27]. Measuring the GCL as a mental process of learning is difficult and requires the measurement of supporting components such as motivation, effort, and metacognitive skills [7]. In a meta-analysis, Lapierre (2022) found that cognitive load measurement is one of the concerns of studies in the field of CLT. He stated that appropriate tools and the use of self-expression are among the concerns of studies in this field [1]. Therefore, it is recommended to use different tools to measure the desired cognitive load component in future studies [5, 17]. Moreover, it is suggested that influential components such as factors affecting the GCL, learning maturity, and educational strategies should be taken into consideration in future studies.

## Conclusion

CLT is a key theory in the purposeful guidance of the process of education, which can guide the educational processes to more effective learning in medical science education systems. The current results showed that CLT had a good fit with the components of memory, ICL, ECL, and clinical decision-making as the key learning outcomes in workplace-based learning in clinical settings. The results showed that the relationship between nursing students’ decision-making skills and extraneous cognitive load is stronger than its relationship with intrinsic cognitive load and memory. Workplace-based learning programs in nursing that aim to improve students’ decision-making skills are suggested to manage extraneous cognitive load by incorporating cognitive load principles into the instructional design of clinical education.

## Data Availability

The datasets generated and/or analyzed during the current study are not publicly available due to the confidentiality of the data of participants but are available from the corresponding author at reasonable request.
